# Inappropriate hospital admission as a risk factor for the subsequent development of adverse events: a cross-sectional study

**DOI:** 10.1186/s12916-023-03024-0

**Published:** 2023-08-17

**Authors:** Diego San Jose-Saras, Jorge Vicente-Guijarro, Paulo Sousa, Paloma Moreno-Nunez, Jesús María Aranaz-Andres, Health Outcomes Research Group of the Instituto Ramón y Cajal de Investigación Sanitaria (IRYCIS), Cristina Díaz-Agero Pérez, Miguel Ignacio Cuchi Alfaro, Juan Manuel Ramos López, Mercedes García Haro, Abelardo Claudio Fernández Chávez, Cornelia Bischofberger Valdés, Amaranta Mcgee Laso, Carmen Garrote Liarte, Gerardo Gómez Montero, Juan Daniel Miranda Cortes, Gema Nieto Gomez, Jessica Alia Herrero, Sara de la Hoz San Clemente, Marta Gonzalez Touya, Moisés David Espejo Mambié, Diana Carretero Gomez, Manuela Serrano Pareja, Marco Antonio Espinel Ruiz, Raquel Gutierrez Gallardo, Eva Elisa Álvarez León, Paloma Navas Gutiérrez, Nerea Armenteros Arzá, Francisco Bolumar Montrull, Ana García de la Santa Viñuela, Raquel Arguedas Sanz, Miriam Roncal Redín

**Affiliations:** 1 Preventive Medicine and Public Health Service, Hospital Universitario Ramón y Cajal, IRYCIS, 28034 Madrid, Spain; 2 Universidad de Alcalá, School of Medicine and Health Sciences, Department of Medicine and Medical Specialities, Alcalá de Henares, Spain; 3 Preventive Medicine and Public Health Service, Hospital Universitario Ramón y Cajal. IRYCIS. CIBER of Epidemiology and Public Health (CIBERESP), 28034 Madrid, Spain; 4 Faculty of Health Sciences, Universidad Internacional de La Rioja, 26006 Logroño, La Rioja Spain; 5 NOVA National School of Public Health, Public Health Research Centre, Comprehensive Health Research Center, CHRC, NOVA University Lisbon, Lisbon, Portugal

**Keywords:** Appropriateness of health care, Inappropriate hospital admission, Patient safety, Adverse events

## Abstract

**Background:**

All health overuse implies an unnecessary risk of patients suffering adverse events (AEs). However, this hypothesis has not been corroborated by direct estimates for inappropriate hospital admission (IHA). The objectives of the study were the following: (1) to analyze the association between IHA and the development of subsequent AEs; (2) to explore the distinct clinical and economic implications of AEs subsequent IHA compared to appropriate admissions.

**Methods:**

An observational cross-sectional study was conducted on hospitalized patients in May 2019 in a high-complexity hospital in Madrid, Spain. The *Appropriateness Evaluation Protocol* was used to measure IHA, and the methodologies of *the Harvard Medical Practice Study* and the *European Point Prevalence Survey of Healthcare-associated Infections* were used to detect and characterize AEs. The association between IHA and the subsequent.

**Results:**

A total of 558 patients in the hospital ward were studied. IHA increased the risk of subsequent occurrence of AEs (OR [95% CI]: 3.54 [1.87 to 6.69], versus appropriate) and doubled the mean AEs per patient (coefficient [95% CI]: 0.19 [0.08 to 0.30] increase, versus appropriate) after adjusting for confounders. IHA was a predictive variable of subsequent AEs and the number of AEs per patient. AEs developed after IHA were associated with scheduled admissions (78.9% of AEs, versus 27.9% after appropriate admissions; *p* < 0.001). Compared with AEs developed after appropriate admissions, AEs after IHA added 2.4 additional days of stay in the intensive care unit and incurred an extra cost of €166,324.9 for the studied sample.

**Conclusions:**

Patients with IHA have a higher risk of subsequent occurrence of AE. Due to the multifactorial nature of AEs, IHA is a possible contributing factor. AEs developed after IHA are associated with scheduled admissions, prolonged ICU stays, and resulted in significant cost overruns.

**Supplementary Information:**

The online version contains supplementary material available at 10.1186/s12916-023-03024-0.

## Background

Health overuse consists of the provision of health services in which the potential harm to the patient exceeds the possible clinical benefit [1]. One form of presentation is inappropriate hospital admission (IHA), a parameter evaluated in the *Appropriateness Evaluation Protocol* (AEP) questionnaire [2]. This validated tool, which was developed by Gertmann and Restuccia [3], has wide international acceptance due to its diagnostic-independent application [2]. In a recent meta-analysis, the frequency of IHA ranged from 8.4 to 17.1% [4], implying a considerable reduction in the availability of hospital beds [5].

Definitions establish that health overuse involves unnecessary risks for patients [6, 7]. These risks can cause adverse events (AEs), defined by *The World Health Organization* as any safety incident derived from health care that causes harm, suffering, disability, or death of a patient [8]. The most accepted methodology for the detection of AEs was developed by Brennan et al. and used in the *Harvard Medical Practice Study *(HMPS) [9]. At the global level, AEs are an important public health problem, as they occur in 10–12% of hospitalized patients [10–12] and approximately 48% of AEs have a moderate-severe impact on the clinical course of patients [13]. In addition, they also represent an important risk for the sustainability of the health system; it has been estimated that AEs could result in an additional cost of more than 1 billion dollars per year [14], and it has been reported that AEs are related to filing claims and the second and third victim phenomenon [15].

Healthcare-associated infections constitute 30–40% of all AE and are associated with the worst healthcare outcomes for the patient in terms of comorbidity and length of hospital stay [16, 17]. For their identification, the *European Centre for Disease Prevention and Control* has developed the specific methodology *European Point Prevalence Survey for healthcare-associated infections* (PPS), which has estimated prevalence values in Europe between 6 and 10% [18, 19].

The assumption that health inappropriateness implies a greater risk for patients is a globally accepted premise [6, 7]. However, this hypothesis has not been fully corroborated by direct estimates. To date, this is the first study that analyzes a possible association between IHA and the subsequent development of AEs, and that calculates the magnitude of this association through direct estimates. In addition, through a review of medical records, this work analyzes whether IHA influences the clinical and economic impacts of AEs. For this, in a pioneering approach, the AEP, HMPS, and PPS methodologies are combined and applied to the same sample, with models adjusted for clinical and epidemiological variables, intrinsic risk factors (IRFs), and extrinsic risk factors (ERFs), not considered in previous studies of the inappropriateness of hospitalization use.

Therefore, this study has the following aims: (1) to analyze if IHA is associated with developing subsequent AEs; (2) to explore the distinct clinical and economic implications of AEs subsequent IHA compared to appropriate admissions.

## Methods

### Design, measurement instruments, and sample selection

This was an observational study with a cross-sectional design. The study setting was a high-complexity hospital with a capacity of 901 beds and 45 operating rooms. Throughout the second week of May 2019, a cross-section of hospitalized patients was obtained for each of the hospital care units. The exact date of the cutoff varied depending on each unit, but the final sample included all patients hospitalized in the center. This work was framed within the Patient Safety Incident Study of Hospitals in the Community of Madrid (ESHMAD) [17, 20], a multicenter patient safety study based on the HMPS methodology [9]. The study, carried out with a cross-sectional design, aimed to include in a subsample the measurement of IHA [5] and, finally, to analyze their association with the subsequent development of AE. Therefore, both were analyzed in the entire subsample. A sample size calculation was not made for this phase due to the lack of previous evidence of the association level of IHA with AEs.

Five validated instruments were used in conjunction with a review of the clinical history of each patient: two versions of the AEP to measure IHA, two tools derived from the *HMPS* methodology to measure and analyze AEs [9], and the PPS [18, 21] as an additional screening for identifying healthcare-associated infections.

1. AEP for admissions [3]: for the measurement of IHA in adult patients. This tool was developed to assess the unnecessary days of hospitalization. The version used includes 16 items related to the clinical status and care needs of the patient to be checked at the moment of admission, which would make it appropriate. If a patient does not meet any of the items, his or her admission is considered inappropriate. The kappa of the tool used was over 0.85 in its validation [22] (Additional file: Table S1).
2. Pediatric adaptation of the AEP (pAEP) [23]: with 22 items for measuring IHA in pediatric patients. The kappa index of the tool used was 0.77 in its validation [24] (Additional file: Table S2).
3. Screening Review Form (SRF): for the screening of AEs from the identification of alert situations in the clinical history. This instrument was developed by the HMPS study [9]. The translated version of the ESHMAD study was used [17, 25]; this version integrated clinical and epidemiological variables from the following studies: ENEAS [10] and IBEAS [16] (Additional file: Table S3). The Screening Review Form had a high sensitivity and negative predictive value in its validation, making it an appropriate screening tool [26].
4. Modular Review Form 2 (MRF2) [27]: for the analysis of the characteristics, types, avoidability, impact, and severity of AEs and additional days of hospital stay related to AEs. This tool was developed by the HMPS [9] to serve as a follow-up instrument for potential AEs identified by the SRF*.* The Spanish versions of the ENEAS [10] and IBEAS [16] studies were used. The kappa index of the tool was 0.61 [9].
5. The European PPS: designed by the *European Centre for Disease Prevention and Control* [18] for identifying healthcare-associated infections. The Prevalence Study of Nosocomial Infections in Spain [21] adapted version was used.

The following exclusion criteria were established: (1) patients who were in the emergency room and who were hospitalized on the same day of the study, as detailed in the PPS [18, 21] and ESHMAD [17, 25] protocols; (2) patients admitted to obstetrics and psychiatric areas, as detailed in the AEP protocol [3]; (3) patients directly admitted to an intensive care unit (ICU), because those patients were not susceptible to meet the IHA criteria of the AEP [3]; and (4) patients in whom AEs were the reason for hospitalization because theoretical models establish that AEs increase the risk of new ones [28] and, according to the main objective of this work, only AEs after admission were considered of interest).

The study was carried out in two sequential phases:

1. Phase 1: cross-section of hospitalized patients, performed during the second week of May 2019. The AEP [3], pAEP [23], SRF [27, 29] and PPS [18, 21] were applied. When the PPS identified healthcare-associated infections, the corresponding AE items of the SRF were checked. The clinical and epidemiological variables of interest were collected. Data were collected by personnel trained in the use of the tools but with no specific skills in Patient Safety or Appropriateness, according to the protocols of the tools used. Data from IHA and the screening were uploaded to two different databases: (1) IHA Database, (2) AEs Database
2. Phase 2: The MRF2 [27, 29] was used if the results of the SRF screening in Phase 1 suggested AEs. False-positive screening results were excluded, and the impact and preventability of confirmed AEs were analyzed. This phase began one month after screening and was only carried out after each patient was discharged. In this phase, data were collected by personnel trained in the use of the tool and with specific training in Patient Safety. They were blinded as they did not know if the patient had an IHA at the moment of review. After this phase, data from both databases were merged.

### Study variables

IHA was any admission in which not a single appropriateness item of the AEP was met. For the confirmation of AEs and their avoidability, the HMPS protocol was used through the MRF2 (scale from 1 to 6; with 1 being 'minimum relationship/evidence' and 6 being ‘practically certain relationship/evidence’; values ≥ 4 were considered positive).

The following clinical and epidemiological variables were collected using the definitions provided in PPS and previous HMPS-based studies (ENEAS [10] and IBEAS [16]): age, sex, type of admission service, Charlson-comorbidity index [30], total stay, reason for discharge, prognosis of the disease that led to admission, IRFs, and ERFs. IRFs were the presence of previous cardiovascular disease, impaired mobility, sensory deficit, diabetes, hypoalbuminemia, immunodeficiency, neutropenia, cirrhosis, coma, previous pressure ulcers, obesity, active smoking, previous neoplasia, and kidney failure. ERFs were the existence of previous surgery, peripheral venous catheter, central venous catheter, urinary catheterization, and mechanical ventilation (Additional file: Tables S3 and S4).

The economic cost due to length of stay related to AEs was calculated from the monetary equivalence for each day of hospital stay for 2019. These data were provided by the accounting department of the study center.

### Statistical analysis

The presence of AEs after hospital admissions was defined as the dependent variable in this study. A descriptive analysis was performed. For qualitative variables, percentages were used; for hypothesis testing, the chi-square or Fisher’s test was used depending on the parametric criteria. For quantitative measures, central measures (mean and median) and dispersion measures were used (standard deviation [SD] and interquartile range [IR]), and comparisons were performed using Student’s *t* test or the Mann‒Whitney *U* test (depending on parametric criteria). The 95% confidence intervals (CI) of means and proportions were estimated. Simple linear regression and univariate logistic regression models were developed.

Two explanatory multivariate models were developed to estimate the association between IHA and subsequent AEs. The first was performed using logistic regression, in which the presence of AEs was the unit of analysis; the second was performed by multiple linear regression, in which the dependent variable was the mean AEs per patient. The confounding variables were studied, considering those that modified the crude association between IHA and the dependent variable by more than 10% [31, 32]. Neither ICU stay nor total stay was included in the analysis because they are intermediate variables between IHA and subsequent AEs. The complete study of the confounding variables can be found in the additional file (Additional file: Tables S5 and S6).

To analyze whether IHA acted as a predictor variable, two predictive models were developed: the first focused on the determinants of the presence of subsequent AEs, and the second focused on the average AEs per patient by logistic multivariate regression and multiple linear regression. Both were developed using a backward modeling strategy with an output *p*-value of 0.100 until finding the most parsimonious model [33]. Overoptimism was corrected with resampling techniques (bootstrap), and goodness of fit was evaluated with the Hosmer‒Lemeshow test and the *R*^2^ statistic.

The statistical analysis was carried out with STATA *Statistical* software, version 16 (StataCorp. 2019. College Station, TX: StataCorp LLC) [34].

#### Ethics committee

As a whole, the different phases of the study were approved by the Ethics Committee of the Hospital (March 19, 2019; reference 057/19) and by the Research Ethics Committee (March 3, 2022; PI reference: 006/2022).

## Results

### Sample characteristics and results tree

At the time of the study, 636 patients were hospitalized. A total of 72 patients did not meet the inclusion criteria: 33 were admitted directly to the ICU, 20 were hospitalized due to a previous AE, and 19 were admitted to psychiatry. There were 6 losses due to patient identification errors.

For the 558 patients analyzed, the mean and median ages were 67.6 (SD: 19.7) and 71 (IR: 57 to 83) years, respectively. There were no relevant differences in the distribution by sex. A total of 50.2%, 47.1%, and 2.7% of patients were admitted to medical, surgical, and pediatric services, respectively. A total of 63.3% of the patients were admitted urgently, 39.1% underwent surgical interventions, and 6.1% died during hospitalization. The mean and median length of stay were 18.2 (SD: 21.8) and 5 (IR: 2 to 12) days, respectively. A total of 94.9% of patients had ≥ 1 IRF, with previous cardiovascular disease (54.7%) and impaired mobility (38.2%) being the most frequent. A total of 95.3% of patients had ≥ 1 ERF, the most frequent being peripheral venous catheters (67.0%) and urinary catheters (21.4%). The characteristics and the bivariate analysis for the sample are shown in Tables 1 and 2.

**Table 1:** Characteristics of the sample

	**Total**	**Patients with AEs developed after the hospital admission**		
	***n* (%)**	***n***	**% (95% CI)**	***p*-value**
**Age**				
Mean (SD)	67.6 (19.7)	73.2 (14.0)	69.7 to 76.7	0.053
Median (IR)	71 (57 to 83)	76 (62 to 84)	-	
**Sex**				
Female	271 (48.6)	30	11.1 (7.6 to 15.4)	0.873
Male	287 (51.4)	33	11.5 (8.0 to 15.8)	
**Type of service**				
Medical specialties	280 (50.2)	36	12.9 (9.2 to 17.4)	0.238
Surgical specialties	263 (47.1)	27	10.3 (6.9 to 14.6)	
Pediatrics	15 (2.7)	0	-	
**Total stay**				
Mean (SD)	18.2 (21.8)	37.9 (35.7)	28.9 to 46.9	< 0.001**
Median (IR)	11 (6 to 23)	24 (11 to 50)	-	
**Inappropriate admission**				
No	487 (87.3)	52	10.7 (8.1 to 13.8)	0.231
Yes	71 (12.7)	11	15.5 (8.0 to 26.0)	
**Type of Admission**				
Urgent	353 (63.3)	41	11.6 (8.5 to 15.4)	0.751
Scheduled	205 (36.7)	22	10.7 (6.8 to 15.8)	
**Surgical intervention**				
Not intervened	340 (60.9)	33	9.7 (6.8 to 13.4)	0.140
Intervened	218 (39.1)	30	13.8 (9.5 to 19.1)	
**Charlson-comorbidity index**				
Mean (SD)	3.0 (2.3)	3.6 (2.3)	3.0 to 4.2	0.019*
Median (IR)	3 (1 to 4)	3 (2 to 5)	-	
**Prognosis of main disease**				
Complete recovery	303 (54.3)	24	7.9 (5.1 to 11.6)	0.043*
Residual disability after episode	166 (29.8)	27	16.3 (11.0 to 22.8)	
Terminal illness	88 (15.8)	12	13.6 (7.2 to 22.6)	
**Number of intrinsic risk factors**				
Absence	39 (7.0)	2	5.1 (0.6 to 17.3)	0.234
1	82 (14.7)	8	9.8 (4.3 to 18.3)	
2	110 (19.7)	9	8.2 (3.8 to 15.0)	
≥ 3	327 (58.6)	44	13.5 (9.9 to 17.6)	
**Number of extrinsic risk factors**				
Absence	128 (22.9)	6	4.7 (1.7 to 9.9)	< 0.001**
1	310 (55.6)	29	9.4 (6.4 to 13.2)	
2	92 (16.5)	18	19.6 (12.0 to 29.1)	
≥ 3	28 (5.0)	10	35.7 (18.6 to 55.9)	
**Death**				
No	524 (93.9)	51	9.7 (7.3 to 12.6)	< 0.001**
Yes	34 (6.1)	12	35.3 (19.7 to 53.5)	
**Total**	558	63	11.3% (8.8 to 14.2)	

*AE*, adverse event; *SD*, standard deviation; *IR*, interquartile range

*P*-value for percentage difference: using chi-square test (parametric) and Fisher’s exact test (nonparametric)

*P*-value for quantitative variables: using the Mann‒Whitney U test when normality criteria were not met

^*^p < 0.05; **p < 0.001

**Table 2:** Distribution of intrinsic and extrinsic risk factors

	**Total**	**Patients with AEs developed after the hospital admission**		
	***n* (%)**^**a**^	***n***	**% (95% CI)**	***p*-value**
**Intrinsic risk factors**				
Cardiovascular disease	305 (54.7)	45	14.8 (10.9 to 19.2)	0.003*
Impaired mobility	209 (37.4)	34	16.3 (11.5 to 22.0)	0.003*
Neoplasia	187 (33.5)	22	11.7 (7.5 to 17.3)	0.743
Hypoalbuminemia	174 (31.2)	32	18.4 (12.9 to 25.0)	< 0.001**
Diabetes	161 (28.9)	21	13.0 (8.3 to 19.2)	0.364
Obesity	127 (22.7)	8	6.3 (2.8 to 12.0)	0.047*
Sensory deficit	126 (22.6)	19	15.1 (9.3 to 22.5)	0.110
Decreased consciousness	123 (22.0)	15	12.2 (7.0 to 19.3)	0.676
Chronic lung disease	90 (16.5)	10	11.1 (5.5 to 19.5)	0.989
Active smoking	86 (15.4)	9	10.5 (4.9 to 18.9)	0.826
Immunodeficiency	41 (7.4)	9	22.0 (10.6 to 37.6)	0.022*
Previous pressure ulcers	30 (5.4)	12	40.0 (22.7 to 59.4)	< 0.001**
Cirrhosis	23 (4.1)	5	21.7 (7.5 to 43.7)	0.163
Neutropenia	18 (3.2)	2	11.1 (1.4 to 34.7)	1.000
Kidney disease	9 (1.6)	1	11.1 (0.2 to 48.2)	1.000
**Extrinsic risk factors**				
Peripheral venous catheter	374 (67.0)	52	13.9 (10.6 to 17.8)	0.010*
Urinary catheter	117 (21.0)	25	21.4 (14.3 to 29.9)	< 0.001**
Central venous catheter	84 (15.1)	17	20.2 (12.3 to 30.4)	0.004*
Mechanical ventilation	7 (1.3)	4	57.1 (18.4 to 90.1)	0.004*
**Total**	558	63	11.3% (8.8 to 14.2)	

*P*-value for percentage difference: chi-square test (parametric) and Fisher’s exact test (nonparametric)

*95% CI*, 95% confidence interval; *AE*, Adverse event

^*^p < 0.05; **p < 0.001

^a^Percentage of patients in the whole sample with the risk factor

**Fig. 1:**
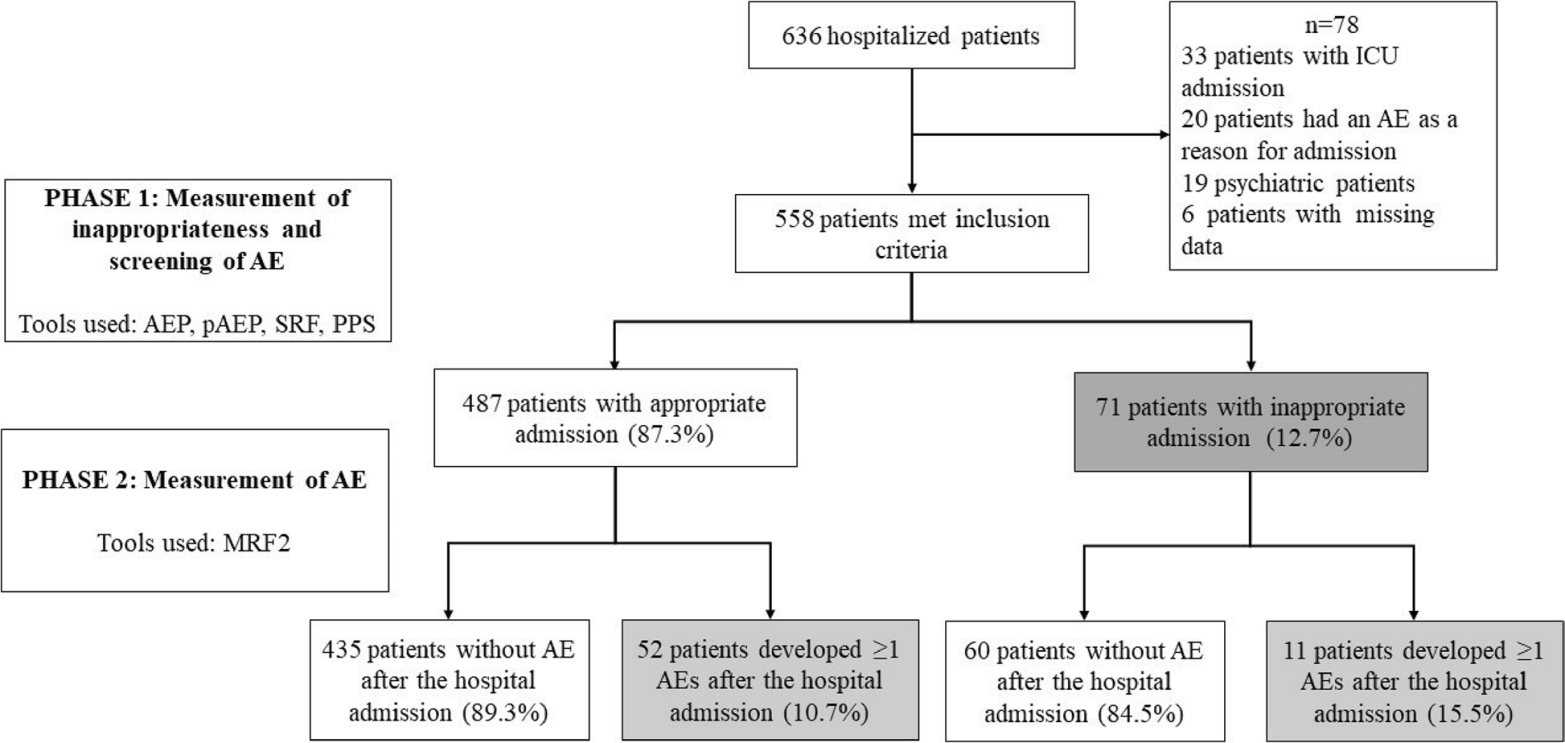
Flowchart for the study. ICU, intensive care unit; AE, adverse events; AEP, Appropriateness Evaluation Protocol; SRF, Screening Review Form; pAEP, Pediatric Appropriateness Evaluation Protocol; PPS, Point Prevalence Survey; MRF2, Modular Review Form 2

A total of 12.7% (71) of the patients were considered IHA, and 11.3% presented ≥ 1 AE (80 AEs in 63 patients). Of the patients with IHA, 15.5% developed ≥ 1 subsequent AEs (10.7% for patients without IHA; *p* = 0.231). Patients with IHA had twice the subsequent AEs than patients with appropriate admission (0.27 AEs per patient versus 0.12 AEs per patient; *p* = 0.015) (Fig. 1).

### Explanatory models of the association between IHA and the subsequent development of AEs

The univariate crude analysis between IHA and the subsequent development of AEs indicated a statistical association (OR [95% CI]: 2.26 [1.26 to 4.04], compared to appropriate admissions). For this model, a higher number of ERFs, the Charlson-comorbidity index, cardiovascular disease, hypoalbuminemia, immunodeficiency, and previous pressure ulcers acted as confounding factors. In the explanatory model, adjusted for confounding variables, the risk of the subsequent development of AEs was 3.54 times higher for patients with IHA than for those with appropriate hospital admissions (OR [95% CI]: 3.54 [1.87 to 6.69]) (Table 3).

**Table 3:** Explanatory models of the association between IHA and the subsequent development of AEs

**Model using logistic regression with AEs as the unit of analysis**	**OR**	**95% CI**	***p*-value**
Crude association between IHA and subsequent development of AEs	2.26	1.26 to 4.04	0.006*
Association between IHA and subsequent AEs, adjusted for confounding variables ^a^	3.54	1.87 to 6.69	< 0.001**
**Model using linear regression of mean AEs with patient as the unit of analysis**	**Coefficient**	**95% CI**	***p*-value**
Crude association between IHA and subsequent AEs	0.14	0.03 to 0.26	0.015*
Association between IHA and subsequent AEs, adjusted for confounding variables ^b^	0.19	0.08 to 0.30	< 0.001**

*IHA*, inappropriate hospital admission; *AE*, adverse event; *OR*, odds ratio; *95% CI*, 95% confidence interval

^*^p < 0.05; **p < 0.001

^a^Model adjusted for confounding variables (number of extrinsic risk factors, Charlson-comorbidity index, cardiovascular disease, hypoalbuminemia, immunodeficiency, and previous pressure ulcers)

^b^Model adjusted for confounding variables (number of extrinsic risk factors, Charlson-comorbidity index, and hypoalbuminemia)

In the crude analysis between IHA and subsequent AEs, with the patient as the unit of analysis, patients with IHA had, on average, 0.14 more subsequent AEs than did patients with appropriate admission. In the multivariate analysis, the number of ERFs, the Charlson-comorbidity index, and hypoalbuminemia acted as confounding factors. This model estimated that IHA increased the average AEs by 0.19 (95% CI: 0.08 to 0.30). Considering that the mean number of subsequent AEs per patient with appropriate admission was 0.12, IHA doubled the average AEs (Table 3).

### Predictive model for AEs developed after hospital admissions

For AEs developed after hospital admissions, IHA acted as a predictor variable in the logistic regression model (OR [95% CI]: 2.29 [1.07 to 4.89], compared with appropriate admission), as did the presence of pressure ulcers (OR [95% CI]: 6.82 [2.87 to 16.2], versus absence), immunodeficiency (OR [95% CI]: 4.75 [1.54 to 14.65], versus absence), central venous catheter (OR [95% CI]: 2.90, versus absence), prior surgical intervention (OR [95% CI]: 2.41 [1.14 to 5.07], versus absence), urinary catheterization (OR [95% CI]: 2.31 [1.41 to 4.37] versus absence), prognosis of residual disability (OR [95% CI]: 2.24 [1.11 to 4.52], versus complete recovery to baseline) and age (OR [95% CI]: 1.02 [1.01 to 1.04] for each 1-year increase) (Table 4).

**Table 4:** Predictive model using logistic regression of factors associated with the development of AEs after hospital admissions

	**Total, *n* (%)**	**AE, *n* (%)**	**Odds Ratio**	**95% CI**	***p*-value**
**Inappropriate admission of patient**					
No	496 (86.5)	61 (12.3)	1.00	-	-
Yes	79 (13.5)	19 (24.1)	2.29	1.07 to 4.89	0.032*
**Age**					
Increase by 1 year, mean in years (SD)	67.9 (19.6)	74.1 (13.3)	1.02	1.01 to 1.04	0.023*
**Sex**					
Female	278 (48.3)	37 (13.3)	1.00	-	-
Male	297 (51.7)	44 (14.5)	1.30	0.74 to 2.30	0.365
**Main disease prognosis**					
Complete recovery	308 (53.5)	28 (9.1)	1.00	-	-
Residual disability	175 (30.4)	36 (20.6)	2.24	1.11 to 4.52	0.024*
Terminal illness	92 (16.0)	16 (17.4)	1.40	0.53 to 3.65	0.498
**Surgical intervention**					
No	346 (60.2)	39 (11.2)	1.00	-	-
Yes	229 (39.8)	41 (17.9)	2.41	1.14 to 5.07	0.021*
**Intrinsic risk factors**					
Cardiovascular disease	318 (55.3)	58 (18.2)	1.96	0.89 to 4.31	0.095
Immunodeficiency	43 (7.5)	11 (25.6)	4.75	1.54 to 14.65	0.007*
Pressure ulcers	37 (6.4)	19 (51.4)	6.82	2.87 to 16.20	< 0.001**
**Extrinsic risk factors**					
Urinary catheter	127 (22.1)	35 (27.6)	2.31	1.21 to 4.37	0.011*
Central venous catheter	98 (17.1)	31 (31.6)	2.90	1.35 to 6.26	0.007*
**Constant**	**-**	**-**	0.00	0.00 to 0.02	< 0.001**

*AE*, adverse event; *95% CI*, 95% confidence interval; *SD*, standard deviation

^*^p < 0.05; **p < 0.001

In the model, sex acted as a control variable due to the existing evidence of its association with AEs developed after hospital admissions. The goodness of fit of the model was evaluated with the Hosmer‒Lemeshow test, obtaining an optimal value (*p* = 0.257).

### Predictive model of the number of AEs developed after hospital admission per patient

In the predictive model developed through multiple linear regression, IHA was a predictor, doubling the mean AEs per patient (coefficient [95% CI]: 0.17 [0.02 to 0.31] increase compared to appropriate admission), as were the presence of pressure ulcers (coefficient [95% CI]: 0.47 [0.17 to 0.77] increase versus absence), central venous catheter (coefficient [95% CI]: 0.24 [0.06 to 0.41] increase versus absence), immunodeficiency (coefficient [95% CI]: 0.17 [0.00 to 0.34] increase versus absence), surgical intervention (coefficient [95% CI]: 0.12 [0.06 to 0.19] increase versus absence), and cardiovascular disease (coefficient [95% CI]: 0.09 [0.02 to 0.16] increase versus absence). The presence of obesity was the only variable that significantly reduced the mean AEs when adjusting for the rest of the variables (coefficient [95% CI]: − 0.09 [− 0.18 to − 0.01] decrease versus absence).

In the goodness of fit analysis, the *R*^2^ statistic was 0.159, with the model having an optimal fit. The final model is presented in the additional file (Additional file: Table S7).

### Impact of the IHA-related AEs

Of the 80 AEs, the most frequent type was healthcare-associated infection (38.8%), followed by procedural complications (26.3%) and nursing care (26.3%). A total of 71.3% of the AEs occurred in the hospitalization ward. A total of 51.2% of the AEs were moderate or severe, and 98.7% required additional health care. The overall preventability of an AE was 69.6%. The mean number of days of additional stay triggered by AEs was 10.3 days in hospitalization wards and 1.5 days in the ICU, with an additional economic cost of €385,238.3.

Compared with AEs developed after appropriate admissions, AEs developed after IHA occurred more frequently among scheduled admissions (78.9%, compared to 27.9% of AEs developed after appropriate admissions; *p* < 0.001) and was associated with more additional days of stay in the ICU (3.3 days on average, compared to 0.9 days for appropriate admissions; p = 0.037). AEs developed after IHA incurred an average cost of €12,600.4 for each additional day of stay and an extra cost of €166,324.9 for all additional days of stay identified. AEs occurring after IHA also incurred higher costs resulting from additional days of ICU stay (€104,475.9, compared to €93,338.5 for AE developed after appropriate admissions; *p* = 0.039) (Table 5).

**Table 5:** AE impact types according to admission appropriateness

	**Total AEs**	**AE developed after appropriate admission**		**AE developed after inappropriate admission**		***p*-value**
	***n* (%)**	***n***	**% (95% CI)**	***n***	**% (95% CI)**	
**Type of AE**						
Health care-associated infections	31 (38.8)	24	39.3 (27.1 to 52.7)	7	36.8 (16.3 to 61.6)	0.218
Complications of a procedure	21 (26.3)	13	21.3 (11.9 to 33.7)	8	42.1 (20.3 to 66.5)	
Complications in care	21 (26.3)	19	31.1 (19.9 to 44.3)	2	10.5 (1.3 to 33.1)	
Negative effects of medication	4 (5.0)	3	4.9 (1.0 to 13.7)	1	5.3 (0.1 to 26.0)	
Other AEs	3 (3.8)	2	3.3 (0.3 to 11.3)	1	5.3 (0.1 to 26.0)	
**Moment of health care in which the AE occurred**						
Related to the admission process	2 (2.5)	2	3.3 (0.3 to 11.3)	0	-	0.103
During a procedure	10 (12.5)	6	9.8 (3.7 to 20.2)	4	21.1 (6.0 to 45.6)	
After the procedure	11 (13.8)	6	9.8 (3.7 to 20.2)	5	26.3 (9.1 to 51.2)	
In the hospital ward	57 (71.3)	47	77.0 (64.5 to 86.8)	10	52.6 (28.9 to 75.6)	
**Severity**						
Mild	39 (48.8)	31	50.8 (37.7 to 63.9)	8	42.1 (20.3 to 66.5)	0.339
Moderate	22 (27.5)	18	29.5 (18.5 to 42.6)	4	21.1 (6.0 to 45.6)	
Severe	19 (23.8)	12	19.7 (10.6 to 31.8)	7	36.8 (16.3 to 61.6)	
**Type of admission**						
Scheduled	32 (40.0)	17	27.9 (17.1 to 40.8)	15	78.9 (54.4 to 93.9)	< 0.001**
Urgent	48 (60.0)	44	72.1 (59.2 to 82.9)	4	21.1 (6.0 to 45.6)	
**Additional assistance as a result of AEs**						
Health care was not affected	1 (1.3)	1	1.6 (0.0 to 8.8)	0	-	0.327
Required only greater observation	3 (3.8)	2	3.3 (0.3 to 11.3)	1	5.3 (0.1 to 26.0)	
Required only 1 additional test	1 (1.3)	1	1.6 (0.0 to 8.8)	0	-	
Medical treatment or rehabilitation	54 (67.5)	44	72.1 (59.2 to 82.9)	10	52.6 (28.9 to 75.6)	
Additional surgical intervention	13 (16.3)	9	14.8 (7.0 to 26.2)	4	21.1 (6.1 to 45.6)	
Intervention or life support treatment	8 (10.0)	4	6.6 (1.8 to 15.9)	4	21.1 (6.1 to 45.6)	
**Impact of AEs on the stay**						
Did not increase the stay	40 (50.0)	32	52.5 (39.3 to 65.4)	8	42.1 (20.3 to 66.5)	0.600
Part of the stay	40 (50.0)	29	47.5 (34.6 to 60.7)	11	57.9 (33.5 to 79.7)	
Avoidability***						
No	24 (30.4)	20	32.8 (21.3 to 46.0)	4	21.2 (6.4 to 47.6)	0.561
Yes	55 (69.6)	41	67.2 (54.0 to 78.7)	14	77.8 (52.4 to 93.6)	
**Death**						
No	65	47	77.0 (64.5 to 86.8)	18	94.7 (74.0 to 99.9)	0.103
Yes	15	14	23.0 (13.2 to 35.5)	1	5.3 (0.1 to 26.0)	
**Additional days of hospitalization triggered by AEs**						
Mean (SD)	10.3 (19.8)	9.4 (19.5)	-	13.2 (20.8)	-	0.481
Median (IR)	0 (0 to 14)	0 (0 to 14)	-	2 (0 to 30)	-	-
Economic cost, daily average per AE	468.7 €/day	383.4 €/day	-	662.7 €/day	-	-
Total sum (days)	822 days	571 days	-	251 days	-	-
Total economic cost	385,238.3 €	218,913.4 €	-	166,324.9 €	-	0.284
**Additional days of ICU stay triggered by AEs**						
Mean (SD)	1.5 (5.3)	0.9 (3.5)	-	3.3 (9.0)	-	0.037*
Median (IR)	0 (0 to 0)	0 (0 to 0)	-	0 (0 to 4)	-	-
Economic cost, daily average per AE	1713.9 €/day	1760.5 €/day	-	1658.4 €/day	-	-
Total sum (days)	116 days	53 days	-	63 days	-	-
Total economic cost	197,864.4 €	93,338.5 €	-	104,475.9 €	-	0.039*
**Total**	80 (100)	61	76.3 (65.4 to 85.1)	19	23.8 (14.9 to 34.6)	

*AE*, adverse events related to health care; *SD*, standard deviation; *IR*, interquartile range

*P*-value for percentage difference: chi-square test (parametric) and Fisher’s exact test (nonparametric)

*P*-value for quantitative variables: Mann‒Whitney *U* test when normality criteria were not met

^*^p < 0.05; **p < 0.001; *** 1 AE excluded for being of unknown avoidability

## Discussion

Approximately 13% of patients presented with IHA, and of these, almost 16% developed ≥ 1 subsequent AEs. After adjusting for confounding variables, patients with IHA had a risk of subsequent occurrence of AEs more than three times higher than that for patients with appropriate admissions. Notably, this is the first study to analyze this association through a direct estimation. IHA acted as a predictor variable of the subsequent development of AEs in both predictive models, using either AE records or patients as a study unit. Compared with AEs developed after appropriate admissions, AEs that occurred after IHA added more than two additional days of stay in the ICU and incurred extra economic costs greater than €160,000 for the total sample studied.

To date, the relationship between health overuse and the risks it entails for patients has been treated mainly from a theoretical framework. There are few studies that have quantified both phenomena using the same sample, and until this study, none had performed a direct estimate of the association between IHA and subsequent AEs. The study was also carried out during May, in a similar way to other studies in the field of epidemiological surveillance, considering this as a representative month of usual clinical practice, not marked by seasonal diseases (such as flu) or by organizational aspects of the hospital (lower care burden) [10, 18, 21].

Previously, indirect estimates were made based on the incidence of AEs related to a procedure and the frequency with which such a procedure is overused. However, these data tended to come from independent studies that did not share the same sample or study population. Brownlee et al. [35] in 2017 carried out an extrapolation of these characteristics. It was estimated that if the frequency of AEs associated with arthroplasties was 7–8% [36] and that 30% of such procedures were indicated unjustifiably [37], approximately 1–2% of arthroplasties would present, simultaneously, overuse and AEs.

To a lesser extent, other more recent studies have made more direct estimates between overuse and AEs. In 2019, Badgery-Parker et al. [38] analyzed hospital-acquired complications after low-value procedures, showing that 26.3% of them were healthcare-associated infections. In Spain, in 2021, AEs derived from the unjustified indication of routine diagnostic and therapeutic procedures in the field of primary care were identified. However, most of these AEs had mild impacts in both adult and pediatric patients [39]. Similarly, other studies have shown that the inappropriate consumption of medications increased the risk of hospitalization by 31% [40].

To date, the only study that explored AEs developed after IHA was that by Canzoniero et al. in 2015 [41], which did so with patients with syncope admitted to a hospital in the USA. This study evaluated the appropriateness of admission using the *San Francisco Syncope Rule* and subsequently analyzed the episodes of hospitalization, finding serious AEs such as hypoglycemia and transfusion errors. However, the study by Canzoniero et al. did not provide a measure of the association between IHA and AEs (as it did not identify the AEs for appropriate admissions) and only investigated a specific pathology. In this regard, our study provides 2 pioneering approaches: (1) it provides a direct estimate of the association between IHA and the subsequent AEs after analyzing AEs in the entire sample, regardless of the appropriateness of admission; and (2) it uses an independent diagnostic tool to measure IHA, providing greater representativeness of the sample and greater external validity of the results.

In our study, the association between IHA and subsequent AEs was analyzed using multivariate explanatory and predictive models and two different units of analysis: (1) based on each patient and (2) based on AE records. This methodology was chosen after observing notable differences in the frequency of the subsequent development of AEs between patients with IHA and patients with appropriate admissions. The most accurate estimation of the association was performed with explanatory models. After adjusting for confounding variables, the model indicated that IHA increased the risk of developing a subsequent AE by more than three times and doubled the mean AE per patient. This finding suggests that reducing IHA would exponentially decrease the burden derived from AEs.

Some hypotheses may explain why IHAs favor the appearance of AEs. One could be that the appearance of an IHA inherently implies an unnecessary increase in hospital stay and the patient's exposure to a high-risk environment, which activates other healthcare errors that lead to the appearance of AEs. In this regard, AEs are not due to a single root cause but to an overlap of failures and errors, including IHA [42].

Another hypothesis would be that IHA could be associated with specific surgical procedures or patient comorbidities that may favor an increment in errors and AEs. In our sample, no specific intervention was found as a possible confounding variable. Furthermore, the AEP has not been used in the analysis of a single intervention because it is diagnostic-independent, so there is no previous evidence of an association between concrete surgeries and IHA [2]. Regarding patient comorbidities, none was found to be associated with IHA in our study. However, this may be due to the sample size, so our findings could serve as a starting point to analyze specific interventions and patients and thus deepen this association with a longitudinal design.

In both predictive models, using patients or AE records as the unit of analysis, IHA was a predictor variable, acting as a contributing factor. This finding has additional importance because the inappropriateness of health care is not considered a contributing factor by most of the tools designed for this purpose, such as the *London Protocol* [28] or the *Systems Engineering Initiative for Patient Safety* model [43]. From this perspective, the results of this study could indicate a need for IHA to be included as a study dimension within the analysis of AEs or sentinel events and could lead to the investigation of other forms of inappropriateness as contributing factors of the AEs.

### AEs after IHA and their impacts

The most frequently identified AEs after IHA were procedural complications and healthcare-associated infections. This result coincides with those of other studies that did not analyze the appropriateness of admission. This was the case in a meta-analysis conducted in 2018, in which procedural complications were the most frequent AEs [44]. For the IBEAS, which used the same AE identification instrument as that used in our study, the most frequent type was healthcare-associated infections, followed by procedural complications [16].

The differences found in our sample indicate that IHA is more frequently associated with subsequent AEs in patients with scheduled admissions. It is previously known that the IHA is associated with scheduled admissions (in this type of patient, the most frequent cause of inappropriateness is due to early admissions; for example, a patient admitted on a Friday for an intervention that is performed on Monday), making this type of patients the most affected by AEs in inappropriate admissions [45].

However, in the explanatory models, the type of admission did not act as a confounder in the association between IHA and AEs. It was not a predictor of AEs in the predictive models either. In addition, in the bivariate analysis, no association was found between AEs and the type of admission (prevalence in scheduled admissions of 10.7% versus 11.6% in urgent ones; *p* = 0.751). These results suggest that the type of admission impacts the type of AE associated, but it is not acting as the cause of this increased association between IHA and AE. In any case, future studies with a longitudinal design should further analyze this association.

A total of 36.8% of AEs developed after IHA had a serious impact on patients, a higher percentage than that obtained in another meta-analysis in 2019 (13.0%) [13]. In addition, compared with the AEs developed after appropriate admissions, AEs developed after IHA led to the ICU stay being extended by more than two additional days.

At the economic level, the average daily cost of AEs developed after IHA was 12,600.4 €/day, which represents a total of 4.6 million € per year for the study hospital. Extrapolating these data to the more than 70 hospitals with more than 500 existing beds in Spain [46], AEs developed after IHA generate an extra cost of €322 million per year for the entire National Health System.

### Limitations

The cross-sectional design of this study has two main limitations: (1) it does not allow establishing causality between IHA and subsequent AEs; and (2) AEs leading to a longer hospital stay could be overrepresented. However, the AEs that lengthen the stay are also those that carry a more serious impact on patients; therefore, their identification and analysis are also more suitable for estimating the association with IHA to prioritize possible strategies for improving patient safety [47]. In addition, the cross-sectional methodology allows a more efficient use of resources [48] because AEs and IHA can be analyzed simultaneously from a combined surveillance system.

Another limitation would be related to the methodology used to measure the IHA. The AEP is the most used tool, and, despite being old, it is still valid, as shown by recent validations in South Korea in 2019 [49] or how it is been used in the analysis of the economic impact in China in the same year [50]. Nevertheless, some criteria of the form should be updated to the current clinical practice in future research, such as 'the administration of treatments by subcutaneous or intramuscular route' or 'the administration of intravenous medication' criteria, which together made appropriate most hospital admissions in our sample. Furthermore, the fact that it is an objective tool that eliminates any judgment of the reviewer, means that accomplish of any criteria could make admissions appropriate without assessing whether it was really necessary. However, these limitations make the tool very specific for detecting inappropriateness by just considering patients who did not receive any therapy or procedure as IHA. Regarding the results of this article, it is possible that these limitations of the form, which make some inappropriate admissions be considered appropriate, acted by underestimating the association between IHA and AEs.’

In addition, it should be considered that, in our study, the association between inappropriate hospital stays and AEs has not been explored. Although the AEP for admissions shares most of the criteria with the AEP for stays, future studies should analyze (1) the association between inappropriate stays and AEs; (2) whether inappropriate stays can be a confounding variable between IHA and AEs.

The study could have benefited from a larger sample size because although statistically significant differences were identified in clinically relevant results, other comparisons needed greater statistical power. An example could be the variable adjustment methodology. The sample size and the lack of previous evidence of what variables could be confounding variables between IHA and AE did not allow us to adjust for more precise techniques. In addition, the comparison between the differences in the consequences of AEs after IHA is limited for this exact reason. Finally, the economic estimates should be interpreted cautiously because they do not include the cost of complementary tests and cascading events derived from IHA or the AEs. Likewise, its extrapolation to other health systems is complex because not all health providers use the exact monetary costs.

### Strengths

This is the first study that provides a direct estimate of the association between IHA and the subsequent occurrence of AEs. This has been possible thanks to identifying AEs not only in IHAs but also in appropriate admissions. In addition, this measure of association was analyzed based on different study units, and models were adjusted for various clinical and epidemiological variables, such as IRFs and ERFs that were not collected or considered in previous appropriateness studies.

It is also a pioneer study because it combines the AEP, HMPS, and PPS methodologies, which include different versions of standardized, validated, and widely accepted measurement instruments. Their joint and simultaneous use is positioned as a new surveillance system in terms of patient safety, capable of analyzing IHA as a possible contributing factor for subsequent AEs. With this, this study allows proposing the hypothesis that IHA and other forms of overuse act as potential causal factors for AEs, which is a possibility that should be specifically studied using longitudinal methodologies.

Similarly, the use of the AEP as a diagnostic-independent application tool provides a high representativeness of the sample and greater external validity of the results. Finally, the application of the HMPS and PPS methodologies has added an additional quality filter in the identification and characterization of AEs.

## Conclusions

Patients with IHA have a risk of subsequent development of AEs more than three times higher than patients with appropriate admissions. Due to the multifactorial nature of AEs, IHA is positioned as a contributing factor.

AEs developed after IHA are associated with scheduled admissions and have greater impacts on both the patient and the health system because extend the stay in the ICU and involve significant economic costs.

The combination of the AEP, HMPS, and PPS methodologies allows the efficient and simultaneous analysis of IHA and subsequent AEs, leading to the establishment of associations between the two. However, longitudinal studies should be conducted to explore the causality between IHA, and other types of overuse, with the subsequent development of AEs by patients.

### Supplementary Information

**Additional file 1: Table S1.** Appropriateness Evaluation Protocol for Admissions. **Table S2.** Pediatric Appropriateness Evaluation Protocol for Admissions. **Table S3.** Definition and applied criteria of variables. **Table S4.** Definition and applied criteria of intrinsic and extrinsic risk factors. **Table S5.** Crude association between inappropriate admissions and the subsequent development of adverse events, by records. **Table S6.** Crude association between inappropriate admissions and the subsequent development of adverse events, per patient. **Table S7.** Predictive model of the number of Adverse Events developed after hospital admissions, per patient.

## Data Availability

The data that support the findings of this study are not openly available due to reasons of sensitivity and are available from the corresponding author upon reasonable request.

## Abbreviations

AE: Adverse events related with health care
AEP: Appropriateness Evaluation Protocol
CI: Confidence interval
ERF: Extrinsic risk factor
ESHMAD: Patient Safety Incident Study of Hospitals in the Community of Madrid
HMPS: Harvard Medical Practice Study
ICU: Intensive care unit
IHA: Inappropriate hospital admission
IR: Interquartile range
IRF: Intrinsic risk factor
MRF2: Modular Review Form 2
OR: Odds ratio
PPS: European Point Prevalence Survey for healthcare-associated infections
SD: Standard deviation
SRF: Screening Review Form
