# Supplementary material for: Inappropriate hospital admission as a risk factor for the subsequent development of adverse events: a cross-sectional study
Source: BMC Med. 2023 Aug 17;21:312. doi: 10.1186/s12916-023-03024-0 (PMC10433586; doi:10.1186/s12916-023-03024-0)
Supplement: Supplementary file 1 — Additional file 1: Table S1. Appropriateness Evaluation Protocol for Admissions. Table S2. Pediatric Appropriateness Evaluation Protocol for Admissions. Table S3. Definition and applied criteria of variables. Table S4. Definition and applied criteria of intrinsic and extrinsic risk factors. Table S5. Crude association between inappropriate admissions and the subsequent development of adverse events, by records. Table S6. Crude association between inappropriate admissions and the subsequent development of adverse events, per patient. Table S7. Predictive model of the number of Adverse Events developed after hospital admissions, per patient. [file 12916_2023_3024_MOESM1_ESM.docx]

# **Inappropriate hospital admission as a risk factor for the subsequent development of adverse events: A cross-sectional study**

Diego San Jose-Saras, PhD ^a,^

Jorge Vicente-Guijarro, PhD ^b,c *^

Paulo Sousa, PhD ^d,e^

Paloma Moreno-Nunez, MD, MPH ^c,f^

Jesús María [Aranaz-Andres](https://www.sciencedirect.com/science/article/pii/S260364792100035X?via%3Dihub#!), PhD ^b,c^

Health Outcomes Research Group of the Instituto Ramón y Cajal de Investigación Sanitaria (IRYCIS)^†^.

^a^ Preventive Medicine and Public Health Service, Hospital Universitario Ramón y Cajal (Ramón y Cajal University Hospital). IRYCIS, 28034 Madrid, Spain. Universidad de Alcalá (University of Alcalá), School of Medicine and Health Sciences, Department of Systems Biology, Alcalá de Henares, Spain.

^b^ Preventive Medicine and Public Health Service, Ramón y Cajal University Hospital. IRYCIS. CIBER of Epidemiology and Public Health (CIBERESP), 28034 Madrid, Spain.

^c^ Faculty of Health Sciences, Universidad Internacional de La Rioja (International University of La Rioja), 26006 Logroño, La Rioja, Spain.

^d^ NOVA National School of Public Health, Public Health Research Center, NOVA University of Lisbon (Universidade NOVA de Lisboa).

^e^ Comprehensive Health Research Center, NOVA University of Lisbon.

^f^ Department of Preventive Medicine and Public Health, Ramón y Cajal University Hospital, IRYCIS, 28034 Madrid, Spain.

*Corresponding author: Jorge de Vicente-Guijarro, Preventive Medicine and Public Health Service, Ramón y Cajal University Hospital. Ctra. de Colmenar Viejo, km. 9,100. 28034. Madrid, Spain. E-mail: [jorge.vicente@salud.maddrid.org](mailto:jorge.vicente@salud.maddrid.org); Phone number: (+34 913 36 83 72)

^†^ Collaborators/Membership of the Health Outcomes Research Group of the Instituto Ramón y Cajal de Investigación Sanitaria (IRYCIS) are listed in Acknowledgments.

# **Supplementary material**

**Table S1: Appropriateness Evaluation Protocol for Admissions**

| **INAPROPRIATENESS OF ADMISSION IN ADULT PATIENTS** | |
| --- | --- |
| **Patient’s clinical situation at admission** | |
|  | 1. Sudden loss of consciousness or disorientation (coma or numbness) |
|  | 2. Pulse < 50 beats or >140 beats per minute |
|  | 3. Blood pressure: Systolic <90 or >200 mmHg; Diastolic <60 or >120 mmHg |
|  | 4. Sudden loss of vision or hearing |
|  | 5. Sudden paralysis of any part of the body |
|  | 6. Persistent fever: 38ºC taken in the mouth (>38.5ºC elsewhere) for more than 5 days. |
|  | 7. Active bleeding |
|  | 8. Severe electrolyte or blood gas disturbances: Na < 123 mEq/l or > 156 mEq/l; K < 2.5 mEq/l or > 6 mEq/l; CO2 combining power (except chronic anomalies < 20 mEq/l CO2 or > 36 mEq/l CO2; arterial pH < 7.30 or > 7.45 |
|  | 9. Electrocardiographic evidence of acute ischemia. |
|  | 10. Suture dehiscence or evisceration. |
| **Clinical services in the first day of hospitalization** | |
|  | 11. Administration of intravenous medication and/or fluid replacement (does not include nasogastric tube for feeding) |
|  | 12. Scheduled/urgent surgery or procedure within the next 24 hours that requires: a) general or regional anesthesia, or b) equipment or materials available only for inpatients |
|  | 13. Vital signs monitoring every 2 hours or more often (including telemetry or cardiac monitoring) |
|  | 14. Chemotherapy that requires continuous observation for the treatment of life-threatening toxic reactions |
|  | 15. Administration of intramuscular antibiotics at least every 8 hours. |
|  | 16. Use of intermittent or continuous respirator, at least every 8 hours |
| **If no box is filled in, admission is inappropriate** | |

**Table S2: Pediatric Appropriateness Evaluation Protocol for Admissions**

| **INAPROPRIATENESS OF ADMISSION IN PEDIATRIC PATIENTS** | |
| --- | --- |
| **Patient’s clinical situation** | |
|  | 1P. Sudden loss of consciousness or disorientation (coma or numbness) |
|  | 2P. Inability to move, feed, breathe, urinate. |
|  | 3P. Acute or progressive sensory or motor, circulatory or respiratory disturbance sufficient to incapacitate the patient. |
|  | 4P.. Acute loss of vision or hearing in the previous 48 hours |
|  | 5P. Difficulty to move any part of the body of acute onset in the previous 48 hours. |
|  | 6P. Persistent fever for more than 5 days (>37.8ºC). |
|  | 7P. Active bleeding |
|  | 8P. Surgical wound dehiscence or evisceration. |
|  | 9P. Severe electrolyte/acid-base disturbance (Na <125 or >156; K < 2.5 OR >5.6; HCO3 <20 or >36; pH <7.3 or >7.45) |
|  | 10P. Hematocrit < 30% |
|  | 11P. HR other than 80-220 (6-23 months), 70-200 (2-6 years), 60-180 (7-11 years), or 50-140 (>12 years) |
|  | 12P. Systolic blood pressure and diastolic blood presure different from (70-120; 40-85 in 6-23 months); (75-125, 40-90 in 2-6 y); (80-130, 45-90 at 7-11 y); (90-200, 60-120 in > 12a). |
|  | 13P. Need for lumbar puncture when it cannot be done on a routine outpatient basis. |
|  | 14P. Any of the following problems that do not respond to outpatient management: seizures, arrhythmia, bornchial asthma or croup, dehydration, encopresis, other problems) |
|  | 15P. Specific pediatric problems: child abuse, noncompliance with therapeutic regimen, special observation or close monitoring of behavior. |
| **Clinical services** | |
|  | 16p. Surgery or procedure scheduled in the next 24 hours that requires general or regional anesthesia or equipment or procedures or facilities available only at the hospital level. |
|  | 17P. ICU treatment |
|  | 18P. Telemetry, cardiac monitoring, or vital signs monitoring every two hours. |
|  | 19p. Intravenous medication and/or fluid replacement (enteral nutrition is not included) |
|  | 20p Chemotherapeutic agents that require continuous observation for possible life-threatening toxic reaction. |
|  | 21p. Intramuscular antibiotics every 8h. |
|  | 22P. Continuous or intermittent ventilator at least every 8 hours, including respiratory physiotherapy. |
| **If no box is filled in, admission is inappropriate** | |
|  |  |

|  |  |
| --- | --- |

| **Table S3: Definition and applied criteria of variables** | | | | | |
| --- | --- | --- | --- | --- | --- |
| **Variable** | **Values** | **Criteria** | **Tool** | **Collection** | **Reference** |
| **AE** | Presence or absence. Scale from 1 to 6: 1 'minimal relationship/evidence' and 6 'practically certain evidence'; values ≥4 were considered positive | Patient safety incident that occurs during medical care and harms a patient, producing an injury, suffering, disability, or death. | MRF2 | Phase 2 | WHO^8^ |
| **Avoidability** | Avoidable or not avoidable. Scale from 1 to 6: 1 'minimal relationship/evidence', and 6 'practically certain evidence'; values ≥4 were considered positive | Avoidability, according to reviewer criteria | MRF2 | Phase 2 | Brennan *et al*^9^ / Woloshynowych *et al*^27^ |
| **Inappropriateness of admission** | Presence or absence; Patient who does not meet any criteria of the AEP or pAEP | Inappropriate hospital admission due to the clinical or healthcare needs of the patient that do not warrant admission to an acute care hospital at the time of occurrence. | AEP / pAEP | Phase 1 | Gertman and Restuccia^3^ |
| **Charlson-comorbidity index** | Numeric | Score of risk of mortality of patients due to their comorbidities | MRF2 | Phase 2 | Charlson et al^30^ |
| **Age** | Numeric | Age of patient in years at admission | SRF | Phase 1 | Brennan *et al*^9^ / PPS^18^ / Aranaz *et al*^10^ |
| **Sex** | Female or Male | Sex of the patient in the clinical record | SRF | Phase 1 | Brennan *et al*^9^ / PPS^18^ / Aranaz *et al*^10^ |
| **Type of admission service** | ‘Medical', 'Surgical' | Type of medical specialty of the admission service | SRF | Phase 1 | Brennan *et al*^9^ / PPS^18^ / Aranaz *et al*^10^ |
| **Prognosis of main disease** | ‘Full recovery', 'Residual disability', or 'Terminal disease' | Prognostic expectation of the diagnosis that produces admission, according to the reviewer | MRF2 | Phase 2 | Brennan *et al*^9^ / Woloshynowych *et al*^27^ |
| **Reason for discharge** | ‘Improvement', 'Transfer' or  'Death' | Administrative cause for hospital discharge | MRF2 | Phase 2 | Brennan *et al*^9^ / Woloshynowych *et al*^27^ |
| **Total days of stay** | Numeric | Total hospital stay in days | MRF2 | Phase 2 | Brennan *et al*^9^ / Woloshynowych *et al*^27^ |
| **Type of AE** | ‘Healthcare-acquired infections', Complications in care', 'Complications of a procedure', 'Adverse effects of medication', and 'Other consequences' | AE type identified by the reviewer. Healthcare-associated infection used PPS criteria. | MRF2 | Phase 2 | Brennan *et al*^7^ / Woloshynowych et al^21^ / PPS^16^ |
| **Severity of AE** | ‘Mild', 'Moderate' or 'Severe'; Mild' – did not affect management or prolong hospital stay; 'Moderate' – stay was prolonged; ‘Severe’ – patient required further surgery, contributed to permanent disability or death | Severity, according to reviewer criteria | MRF2 | Phase 2 | Brennan *et al*^7^ / Woloshynowych *et al*^21^ |
| **Time of appearance of the AE** | ‘Related to the admission process', 'During a procedure', 'After the procedure' or 'On the hospitalization floor' | Point of healthcare in which the AE appeared | MRF2 | Phase 2 | Brennan *et al*^9^ / Woloshynowych *et al*^27^ |
| **Stay days added by AEs** | Numeric | Days of hospital stay added because of AEs | MRF2 | Phase 2 | Brennan *et al*^9^ / Woloshynowych *et al*^27^ |
| **ICU stay days added by AEs** | Numeric | ICU stay days added because of AEs | MRF2 | Phase 2 | Brennan *et al*^9^ / Woloshynowych *et al*^27^ |
| AE: Adverse Event; ICU: Intensive Care Unit; MRF2: Modular Review Form 2; WHO: World Health Organization; AEP: Appropriateness Evaluation Protocol; pAEP: Pediatric Appropriateness Evaluation Protocol; SRF: Screening Review Form; PPS: Prevalence Point Survey of Healthcare-associated infections and antimicrobial use in European acute care hospitals. | | | | | |

| **Table S4. Definition and applied criteria of intrinsic and extrinsic risk factors** | | | | | |
| --- | --- | --- | --- | --- | --- |
| **Definition of intrinsic risk factors** | | | | | |
| **Variable** | **Values** | **Criteria** | **Tool** | **Collection** | **Reference** |
| **Kidney Failure** | Presence or absence | Clinical diagnosis prior admission or creatinine values higher than 1.7 mg/dl at admission | SRF | Phase 1 | PPS^18^ / Aranaz *et al*^10^ |
| **Sensory deficit** | Presence or absence | Blindness or deafness before admission | SRF | Phase 1 | PPS^18^ / Aranaz *et al*^10^ |
| **Impaired mobility** | Presence or absence | Difficulty in mobility on admission | SRF | Phase 1 | PPS^18^ / Aranaz *et al*^10^ |
| **Immunodeficiency** | Presence or absence | Primary or secondary immunodeficiency before admission | SRF | Phase 1 | PPS^18^ / Aranaz *et al*^10^ |
| **Neutropenia** | Presence or absence | Neutrophil count <1000 | SRF | Phase 1 | PPS^18^ / Aranaz *et al*^10^ |
| **Liver cirrhosis** | Presence or absence | Clinical diagnosis before admission | SRF | Phase 1 | PPS^18^ / Aranaz *et al*^10^ |
| **Cardiovascular disease** | Presence or absence | Clinical diagnosis of any disease before admission | SRF | Phase 1 | PPS^18^ / Aranaz *et al*^10^ |
| **Neoplasia:** | Presence or absence | Diagnosis in the previous 5 years | SRF | Phase 1 | PPS^18^ / Aranaz *et al*^10^ |
| **Chronic obstructive pulmonary disease** | Presence or absence | Clinical diagnosis | SRF | Phase 1 | PPS^18^ / Aranaz *et al*^10^ |
| **Pressure ulcers** | Presence or absence | Presence before admission | SRF | Phase 1 | PPS^18^ / Aranaz *et al*^10^ |
| **Obesity** | Presence or absence | BMI >30 | SRF | Phase 1 | PPS^18^ / Aranaz *et al*^10^ |
| **Active smoking** | Presence or absence | At admission | SRF | Phase 1 | PPS^18^ / Aranaz *et al*^10^ |
| **Definition of extrinsic risk factors** | | | | | |
| **Variable** | **Values** | **Criteria** | **Tool** | **Collection** | **Reference** |
| **Previous surgery** | Presence or absence | Surgical intervention before screening day | SRF | Phase 1 | PPS^18^ / Aranaz *et al*^10^ |
| **Peripheral vascular catheter** | Presence or absence | Present at screening | SRF | Phase 1 | PPS^18^ / Aranaz *et al*^10^ |
| **Central vascular catheter** | Presence or absence | Present at screening | SRF | Phase 1 | PPS^18^ / Aranaz *et al*^10^ |
| **Urinary catheterization** | Presence or absence | Present at screening | SRF | Phase 1 | PPS^18^ / Aranaz *et al*^10^ |
| **Intubation** | Presence or absence | Present at screening | SRF | Phase 1 | PPS^18^ / Aranaz *et al*^10^ |
| MRF2: Modular Review Form 2; WHO: World Health Organization; AEP: Appropriateness Evaluation Protocol; pAEP: Pediatric Appropriateness Evaluation Protocol; SRF: Screening Review Form; PPS: Prevalence Point Survey of Healthcare-associated infections and antimicrobial use in European acute care hospitals | | | | | |

| **Table S5. Crude association between inappropriate admissions and the subsequent development of adverse events, by records** | |
| --- | --- |
| Coefficient | **0.816** |
| Adjusted for age | 0.805 |
| Adjusted for sex | 0.816 |
| Adjusted for the number of intrinsic risk factors | 0.850 |
| Adjusted for the number of extrinsic risk factors | 0.987* |
| Adjusted for Charlson-comorbidity index | 0.912* |
| Adjusted for prognosis of main illness | 0.850 |
| Adjusted for type of service | 0.872 |
| Adjusted for type of admission | 0.809 |
| Adjusted for surgical intervention | 0.759 |
| Adjusted for Chronic kidney disease | 0.844 |
| Adjusted for sensory deficit | 0.852 |
| Adjusted for impaired mobility | 0.770 |
| Adjusted for hypoalbuminemia | 1.024* |
| Adjusted for diabetes | 0.846 |
| Adjusted for obesity | 0.874 |
| Adjusted for smoking | 0.873 |
| Adjusted for cardiovascular disease | 0.912* |
| Adjusted for neoplasia | 0.868 |
| Adjusted for chronic lung disease | 0.847 |
| Adjusted for cirrhosis | 0.832 |
| Adjusted for coma | 0.851 |
| Adjusted for neutropenia | 0.853 |
| Adjusted for immunodeficiency | 0.908* |
| Adjusted for pressure ulcers | 0.931* |
| Adjusted for urinary catheterization | 0.860 |
| Adjusted for peripheral venous catheter | 0.792 |
| Adjusted for central venous catheter | 0.822 |
| Adjusted for mechanical ventilation | 0.837 |
| * Confounding variables |  |

| **Table S6. Crude association between inappropriate admissions and the subsequent development of adverse events, per patient** | |
| --- | --- |
| Coefficient | **0.142** |
| Adjusted for age | 0.140 |
| Adjusted for sex | 0.142 |
| Adjusted for the number of intrinsic risk factors | 0.147 |
| Adjusted for the number of extrinsic risk factors | 0.174* |
| Adjusted for Charlson-comorbidity index | 0.156* |
| Adjusted for prognosis of main disease | 0.143 |
| Adjusted for type of service | 0.147 |
| Adjusted for type of admission | 0.141 |
| Adjusted for surgical intervention | 0.138 |
| Adjusted for chronic kidney disease | 0.147 |
| Adjusted for sensory deficit | 0.146 |
| Adjusted for impaired mobility | 0.138 |
| Adjusted for hypoalbuminemia | 0.164* |
| Adjusted for diabetes | 0.148 |
| Adjusted for obesity | 0.150 |
| Adjusted for smoking | 0.151 |
| Adjusted for cardiovascular disease | 0.155 |
| Adjusted for neoplasia | 0.151 |
| Adjusted for chronic lung disease | 0.148 |
| Adjusted for cirrhosis | 0.146 |
| Adjusted for coma | 0.150 |
| Adjusted for neutropenia | 0.149 |
| Adjusted for immunodeficiency | 0.153 |
| Adjusted for pressure ulcers | 0.155 |
| Adjusted for urinary catheterization | 0.155 |
| Adjusted for peripheral venous catheter | 0.133 |
| Adjusted for central venous catheter | 0.153 |
| Adjusted for mechanical ventilation | 0.139 |
| *Confounding variables |  |

| **Table S7. Predictive model of the number of Adverse Events developed after hospital admissions, per patient** | | | | | | |
| --- | --- | --- | --- | --- | --- | --- |
|  | **Total patients, n (%)** | **Patients with AEs, n (%)** | **Mean AEs (SD)** | **Coefficient** | **95% CI** | **p-value** |
| **Inappropriate admission** | | | | | | |
| No | 487 (87.3) | 52 (10.7) | 0.12 (0.39) | 0.00 | - | - |
| Yes | 71 (12.7) | 11 (15.5) | 0.27 (0.76) | 0.17 | 0.02 to 0.31 | 0.027* |
| **Age** | | | | | | |
| Increase by 1 year, mean in years (SD) | 67.6 (19.7) | 73.2 (14.0) | - | 0.01 | 0.00 to 0.01 | 0.022* |
| **Sex** | | | | | | |
| Female | 271 (48.6) | 30 (11.1) | 0.14 (0.45) | 0.00 | - | - |
| Male | 287 (51.4) | 33 (11.5) | 0.15 (47.5) | 0.01 | -0.05 to 0.08 | 0.672 |
| **Surgical intervention** | | | | | | |
| No | 340 (60.9) | 33 (9.7) | 0.11 (0.39) | 0.00 | - | - |
| Yes | 218 (39.1) | 30 (13.8) | 0.19 (0.55) | 0.12 | 0.06 to 0.19 | <0.001** |
| **Main disease prognosis** | | | | | | |
| Complete recovery | 303 (54.4) | 24 (7.9) | 0.09 (0.35) | 0.00 | - | - |
| Residual disability after episode | 166 (29.8) | 27 (16.3) | 0.22 (0.59) | 0.08 | -0.00 to 0.17 | 0.058 |
| Terminal illness | 88 (15.8) | 12 (13.6) | 0.18 (0.49) | 0.01 | -0.10 to 0.13 | 0.815 |
| **Intrinsic risk factors** | | | | | | |
| Cardiovascular disease | 305 (55.8) | 45 (14.8) | 0.19 (0.53) | 0.09 | 0.02 to 0.16 | 0.016* |
| Obesity | 127 (23.2) | 8 (6.3) | 0.09 (0.38) | -0.09 | -0.18 to -0.01 | 0.030* |
| Immunodeficiency | 41 (7.5) | 9 (22.0) | 0.18 (0.40) | 0.17 | 0.00 to 0.34 | 0.046* |
| Pressure ulcers | 30 (5.5) | 12 (40.0) | 0.63 (0.99) | 0.47 | 0.17 to 0.77 | 0.002* |
| **Extrinsic risk factors** | | | | | | |
| Urinary catheter | 117 (21.4) | 25 (21.4) | 0.30 (0.62) | 0.11 | -0.02 to 0.23 | 0.099 |
| Central venous catheter | 84 (15.4) | 17 (20.2) | 0.37 (0.88) | 0.24 | 0.06 to 0.41 | 0.008* |
| **Constant** | **-** | **-** | **-** | -0.37 | -0.37 to -0.06 | 0.007* |
| Multivariate analysis using linear regression. The influence of the variables on the mean number of AEs per patient is analyzed. Model with greatest parsimony, adjusted for inappropriateness, surgical intervention, and intrinsic and extrinsic risk factors  95% CI: 95% confidence interval; AE: adverse event; SD: standard deviation  * p<0.05; ** p<0.001 | | | | | | |
